# Examining the relation between self-realization and burnout levels of nurses

**DOI:** 10.1097/MD.0000000000030592

**Published:** 2022-10-07

**Authors:** Senay Cetinkaya, N. Ecem Oksal Gunes

**Affiliations:** a Faculty of Health Sciences, Department of Nursing, Child Health and Nursing, Çukurova University, Adana, Turkey; b Çukurova University, Health Sciences Institute, Nursing Care Main Branch of Science, Adana, Turkey.

**Keywords:** burnout, nurse, self-realization

## Abstract

Self-realization involves the realization of one’s aims, revealing his/her potential, self-satisfaction, personal accomplishment, and scientific inventions. It is important to know nurses’ self-realization levels and the variables that affect it, because self-fulfillment is necessary for the personal and professional development of nurses. This study was conducted to determine the relation between self-realization and burnout levels of nurses. The study is a cross-sectional study that was conducted on 136 nurses who worked at Çukurova University, Faculty of Medicine Hospital in Adana, Turkey. The data were collected with the Personal Information Form and the “Self-realization” sub-dimension of the “Healthy Lifestyle Behaviors Scale” and the “Maslach Burnout Scale.” The analyses of the data were made in the SPSS demo packet Program with percentages, averages, variance analysis, Student *t* test, and Mann–Whitney *U* test. The Ethical Board Approval and Institutional Approval were obtained for the study. The self-realization score average of the nurses was 32.93 ± 6.43. The self-realization scores of the nurses who preferred their jobs willingly were found to be higher, and their burnout levels were found to be lower. It was also determined that female nurses experienced more emotional burnout compared to male nurses. In the present study, it was determined that the personal accomplishment score averages were higher and the desensitization and emotional burnout score averages, which are among the burnout scale sub-dimensions, were lower in those who had higher self-realization scores.

## 1. Introduction

The most important factor directing human behavior is the *need*. According to Maslow, if the needs mentioned in the first 4 steps are met in a respective order, the individual is directed to meet the self-actualization need, which is in a higher level.^[1]^ Self-realization is one of the basic functions of humans. Self-realization needs involve the realization of one’s aims, revealing his/her potential, personal satisfaction, personal accomplishment (PA), and scientific inventions.^[2,3]^

Individuals want to make choices, make decisions, use skills, solve problems, and make contributions on results that may be significant for them;^[4]^ however, excessive control is not a desired outcome as well as the lack of control. Because excessive control may cause that individuals feel exhausted, weariness in their work, think that they are inadequate, and experience decreases in terms of self-confidence.^[5]^ The scientific and technological developments have influenced the healthcare system and the services related to it.^[6]^ These developments make it compulsory for nurses to become professional members by improving themselves in a continuous and individual manner, by taking responsibilities, having leadership characteristics, producing solutions for problems through research, and establishing strong communications with other people.^[7]^ These characteristics show the importance of reaching the self-realization step by nurses in the hierarchy of basic needs.

“*The opportunity of being able to make choices and decisions, solve problems and to fulfill duties assigned*,” which an individual has over his/her job is defined as “control.”^[8]^ The adaptation of an individuals with their work appear when their control overlaps with the responsibility at work.^[9]^ The disagreement between the individuals and their work in terms of control appears when individuals are not able to control the resources that are necessary for their work, or if they do not have the authority for making decisions about the way the work is done. This disagreement causes that individuals move away from their jobs, their performance decreases, and eventually, face burnout. The disagreement in terms of control is related to the decrease of the burnout in PA.^[8]^

A great number of studies have been conducted on the burnout concept. However, the most common and approved definition of burnout was made by Christina Maslach, who also developed the Maslach Burnout Scale (MBS). According to Maslach, “*Burnout is a syndrome that occurs when the physical and long-term weariness, helplessness and hopelessness in people, who are exposed to constant and intense emotional demands and who have to work and interact with other people in their jobs, are reflected to the work done, to the life and to other people with negative attitudes*.”^[10]^ Maslach defined burnout as “*the emotional burnout, desensitization and low personal accomplishment seen in individuals who are in intense relation with people due to their work*.”^[8,11]^ Dall’Ora et al found that the associations hypothesized by Maslach’s theory between mismatches in areas of work-life and burnout were generally supported.^[12]^

When nurses, who have active roles in all areas of healthcare services, are satisfied with their work and when they are happy and productive, this will affect the quality of the service they provide, that is, “*the return*.” Job satisfaction was defined as the level of covering the physical, psychological, and social needs of employees in accordance with their expectations.^[13]^

Research consistently found that adverse job characteristics – high workload, low staffing levels, long shifts, low control, low schedule flexibility, time pressure, high job and psychological demands, low task variety, role conflict, low autonomy, negative nurse-physician relationship, poor supervisor/leader support, poor leadership, negative team relationship, and job insecurity – were associated with burnout in nursing.^[12]^ The nursing shortage is a significant issue affecting healthcare systems globally.^[14]^

A nurse having job satisfaction will satisfy the self-realization need. A nurse with self-realization will be able to reduce the burnout level to the lowest level as a nurse developing in individual and professional terms, taking responsibilities, producing solutions to problems by research, having leadership skills and strong interpersonal communication. In this respect, it is important to know the relation between the self-realization and burnout levels of nurses.

## 2. Materials and Methods

The present study is a cross-sectional study that was conducted for the purpose of examining the relation between self-realization levels of the nurses and their burnout levels. No research on this subject was found in the literature review.

### 2.1. Population and sampling

The study population consisted of 830 nurses who worked in a hospital in Adana, Turkey. No sampling selection was made in the present study. The purpose of the study was explained to the nurses by the researcher. According to a similar study, the minimum sampling size was calculated as 86.^[15]^ The study was conducted with 136 nurses (16.38%) who accepted to participate in the study.

### 2.2. Data collection tools

The data of the study were collected by using the Personal Information Form, the “Self-realization Sub-dimension” of the “Healthy Lifestyle Behaviors Scale” and the “Maslach Burnout Scale” (i.e., Maslach Burnout Inventory), which is used commonly in the literature after being adopted into Turkish with validity and reliability study by Çapri in 2006 following its development by Christina Maslach and Susan Jackson to measure the burnout levels of the participants.

### 2.3. Personal information form

The personal information form, which was developed upon a literature review, included 17 questions that might affect the self-realization and burnout levels of the nurses such as the socio-demographic and professional characteristics, professional experience years, the duty in the department, reason for choosing this profession, love for the profession, approximately daily working hours, total service years in profession, how the profession was chosen, membership status of any association, participation to any course/congresses, and professional future plans.

### 2.4. The “self-realization” sub-dimension of the healthy lifestyle behaviors scale

The “Self-realization” sub-dimension of the “Healthy Lifestyle Behaviors Scale,” which was developed by Pender (1987) as consisting of 13 questions and which was studied in terms of validity and reliability in Turkish version by Esin (1997) in the form of a 4-point Likert type, was employed.^[16]^ The lowest score that might be received from the scale is 13, and the highest score is 52. As the self-realization score received from the scale increases, the self-realization level also increases. In the study that was conducted by Esin (1997), the Cronbach Alpha Reliability Coefficient of the Self-Realization Sub-Dimension was reported as 0.77.

### 2.5. MBS (Maslach Burnout Inventory)

The MBS that was developed by Maslach and Jackson in 1981 was employed to measure the burnout levels of the academic personnel.^[10]^ The validity and reliability study of the Turkish version of the scale was conducted by Çapri in 2006.^[15]^ Çapri (2006), who conducted the Turkish adaptation study of the scale, reported the Cronbach Alpha Reliability Coefficient of the scale as 0.93. The scale consists of 5 options. According to their severity levels, these options are “0-never, 1-very rarely, 2-sometimes, 3-most of the time, 4-always.” Consisting of 22 expressions, this scale measures the burnout status in 3 different dimensions. The first one of these is the emotional burnout (EB) and consists of 9 statements; the second one is the Desensitization (i.e., Depersonalization) and consists of 5 statements; and the last one is the PA sub-dimension and consists of 8 statements. Contrary to the others, the PA statements given in the questionnaire are positive expressions, and high scores received in these expressions means high PA; on the other hand, they also show that the burnout level is low. In this respect, high scores received from the EB scale and from the Desensitization sub-scale, and low scores received from PA sub-scales express high burnout levels.^[15]^

### 2.6. The collection of the data

The data of the study were collected with the Questionnaire Form between March 1 and May 1, 2018. The researchers went to the clinics during the day shift and explained the purpose of the study to the Clinical Attendant Nurses, and left questionnaire forms as many of the number of the nurses who worked at the clinic. One week later, the researchers went to the clinics again and collected the forms. The answering time for the questionnaire was approximately 8 to 10 minutes.

### 2.7. The analysis of the data

The SPSS demo packet program was employed to analyze the study data. The descriptive, parametric, and non-parametric statistical analyses were employed in these analyses. The mean, standard deviation, median, frequency, percentage, minimum, and maximum values were computed in the descriptive statistical analyses. The relations between the data on socio-demographic variables, the profession, and the self-realization scale results were analyzed with the Mann–Whitney *U* test, variance analysis, Student *t* test, and Bonferroni Multiple Comparison Test. The relation between the burnout levels and performance scores of the nurses was evaluated with the Pearson Correlation Test. The *P* value < .05 was accepted as the statistically significant limit.

### 2.8. The ethical aspect of the study

The required approvals for the scales that were used in collecting the study data were obtained. The approval of the institution and the approval of the Ethical Board of Çukurova University, Faculty of Medicine were also obtained.

### 2.9. The limitations of the study

The fact that half of the nurses did not want to participate in the study is the limitation of it.

## 3. Results

The socio-demographic characteristics of the nurses, who participated in the present study, the data on their profession, their self-realization and total score averages, and the burnout levels are presented in this section.

### 3.1. The socio-demographic characteristics of the nurses

The average age of the nurses was 32.18 ± 5.6. A total of 42 (30.9%) of them were between 18 and 24 years of age, 18 (13.2%) were between 25 and 29, 23 (16.9%) were between 30 and 34, 53 (39%) were between 35 and above years of age. A total of 118 (86.8%) of the nurses were female, 18 (13.2%) were male; and 86 (63.2%) were married, 45 (33.1%) were single; and 4 (2.9%) were divorced. A total of 80 (58.8%) of the nurses had children, and 56 (41.2%) did not have any children. According to the rates of those who responded to this part of the questionnaire, 14 (26.9%) had 1 child; 32 (61.6%) had 2 children, and 6 (11.5%) had 3 or 4 children. When the income levels were questioned, it was determined that 18 (13.6%) had good economic status, 104 (78.8%) had moderate economic status, and 10 (7.6%) had bad economic status. A total of 40 (29.4%) of the nurses were graduated from Vocational Health High School, 19 (14%) were Associate Degree holders, 73 (53.7%) were Undergraduate Degree holders, and 4 (2.9%) were Postgraduate Degree holders.

The distribution of the findings on the professions of the nurses who participated in the study is given in Table 1. A total of 16.9% of the nurses had 0 to 4 years of professional experience (Table 1), and the average professional experience was 11.65 ± 6.5 years.

**Table 1:** Professional characteristics of the nurses who participated in the study.

Variables and groups	n	%
Did they choose their profession willingly?		
Yes	65	47.8
No	70	51.5
Reason for preferring the profession		
Loves the profession	33	24.6
The desire of the family	46	34.3
Financial gains	40	29.9
Other	15	11.2
How many years has she/he been working as a nurse?		
0–4	23	16.9
5–9	47	34.6
10–19	40	29.4
20–35	26	19.1
How many years has she/he been working in this department?		
0–4	41	30.6
5–9	54	40.3
10–19	27	20.1
20–35	12	9.0
Does she/he like the profession?		
Yes	103	78.6
No	28	21.4
Has she/he received any training to work in this department?		
Yes	60	44.4
No	75	55.6
Has she/he asked to work in this department?		
Yes	46	34.1
No	89	65.9
Are they satisfied from working in their current department?		
Yes	94	70.7
No	39	29.3
How many patients (approximately) are dealt with in each duty?		
0–4	18	15.7
5–9	17	14.8
10–19	56	48.7
20–40	24	20.9
Duty in the department where she/he works		
Clinical nurse	77	57.0
Attendant nurse	22	16.3
Intensive care nurse	24	17.18
Other	12	8.9
Does she/he have a membership at a National Professional Association?		
Yes	23	17.0
No	112	83.0
Does she/he participate in professional courses/congresses?		
Yes	97	72.4
No	37	27.6
What are the future plans about profession?		
Completing undergraduate study	14	8.8
Postgraduate study	39	24.5
Becoming an academician	11	6.9
Improving myself to be better in my profession	66	41.5
I do not have any future plans	29	18.2
Ideas about the future of the profession		
Positive	63	52.1
Negative	58	47.9
If the ideas about the future of the profession are positive, why?		
Educational level has increased	12	85.7
The transition of males into the profession	2	14.3
If the ideas about the future of the profession are negative, why?		
Due importance is not given for the profession	19	50.0
The workload is too much	9	23.7
Uncertain job description	4	10.5
Lack of employees	2	5.3
Training is not adequate	3	7.9
Being considered as assisting employees	1	2.6

A total of 34.1% of the nurses stated that they worked at their department with their own desires, 44.4% stated that they received some kind of training to work at their departments, and 70.7% stated that they were happy to work in their departments. It was determined that most of the nurses (57%) worked as clinical nurses, 17% had memberships at national associations, and 72.4% participated in courses/congresses (Table 1).

In Table 2, it is shown that the lowest average score of the nurses in Self-realization Scale was determined in the 12th item, which said “*I am proud of my accomplishment*”; the highest average score was determined in the 5th Item, which said “*I know my weak and strong sides*.” The self-realization score average of the nurses who constituted the sampling group was determined as 32.93 ± 5.8 (min.: 25; max.: 52).

**Table 2:** The items of the self-realization scale of the nurses and total score averages (n = 136).

	Self-realization scale items	*X* ± SD
1	I am optimistic	2.90 ± 0.72
2	I care about myself	2.82 ± 0.78
3	I am generally happy	2.65 ± 0.76
4	I am aware of the positive development in my personality	3.07 ± 0.70
5	I know my strong and weak sides	3.34 ± .65
6	I consider the future in my works	2.82 ± 0.85
7	I am hopeful and optimistic about the future	2.63 ± 0.82
8	I am aware of the things that are important for me	2.93 ± 0.79
9	I spend adequate time for my job and entertainment	2.49 ± 0.85
10	I consider my targets in a rationalist manner	2.76 ± 0.81
11	I consider everyday worth living	2.73 ± 0.83
12	I am proud if my accomplishment	2.32 ± 0.94
13	I consider the environment in which I live worth living	2.60 ± 0.79
	Total	32.93 ± 6.43

SD = standard deviation.

In Table 3, it is shown that the EB sub-dimension score average was 19.81 ± 6.99, desensitization sub-dimension score average was 7.07 ± 3.75, PA sub-dimension score average was 19.65 ± 4.87, and self-realization score average was 32.93 ± 5.8 (min.: 25; max.: 52) for the nurses in the present study, which was conducted to determine the burnout levels of the nurses who worked in a hospital in Adana.

**Table 3:** The average of the burnout sub-scales and its standard deviation.

Emotional burnout	Desensitization	Personal accomplishment	Self-realization
19.81 ± 6.99	7.07 ± 3.75	19.65 ± 4.87	32.93 ± 6.43

As a result of the analyses of the data, no significant differences were detected between the age, marital status, number of children and their plans about the future of their profession variables of the nurses, and their “self-realization” and “burnout scale sub-dimension” score averages (*P* > .05). However, the difference was significant between the income status of the nurses, who chose their profession willingly, the reason for choosing their profession, the love for their profession, satisfaction with working in their departments, the number of the patients they look after, participation to courses/congresses, ideas about the future of their professions, and their self-realization score averages (*P* < .05). The self-realization score averages of the nurses who had good income status, who chose their profession willingly, who were satisfied from working in their departments, and who participated in courses/congresses were found to be higher. It was determined that the self-realization score averages of the nurses, who had positive ideas about the future of their professions, and who worked as Attendant Nurses in their departments were higher (Table 4).

**Table 4:** Comparison of the self-realization scores and burnout sub-scale scores of the nurses with their socio-demographic characteristics.

Variables	Self-realization	Desensitization	Personal accomplishment	Emotional burnout
**Did they choose their profession willingly?**				
Yes	37.65 ± 6.95	6.17 ± 3.81	20.71 ± 5.09	18.22 ± 6.86
No	33.67 ± 6.68	7.89 ± 3.56	18.66 ± 4.51	21.26 ± 6.88
*T* calculation	3.38	2.71	2.47	2.56
*P*	.001*	.008*	.01*	.01*
**Reason for preferring the profession**				
Loves the profession	38.33 ± 7.41	5.33 ± 2.86	20.27 ± 5.51	16.03 ± 5.63
The desire of the family	34.96 ± 5.75	7.85 ± 3.58	20.02 ± 5.06	20.85 ± 7.30
Financial gains	35.33 ± 7.72	8.13 ± 4.11	19.00 ± 4.41	22.08 ± 6.36
Other	32.47 ± 6.71	6.01 ± 3.41	19.00 ± 4.12	19.33 ± 7.80
*F* calculation	2.91	4.92	0.58	5.39
*P*	.03*	.003*	.62	.002*
**Does s/he like the profession?**				
Yes	36.95 ± 6.50	6.46 ± 3.63	20.52 ± 4.64	18.39 ± 6.57
No	31.18 ± 5.96	9.46 ± 3.55	16.71 ± 4.36	24.57 ± 6.73
*t* calculation	4.23	3.91	3.89	4.38
*P*	.01*	.02*	.01*	.03*
**How many years has s/he been working as a nurse?**				
0–4	36.04 ± 7.46	6.87 ± 3.18	17.70 ± 6.27	17.09 ± 7.25
5–9	34.55 ± 6.57	8.64 ± 3.86	19.68 ± 4.03	21.28 ± 7.33
10–19	35.00 ± 7.91	6.48 ± 3.85	19.13 ± 4.95	20.80 ± 5.78
20–35	37.85 ± 5.85	5.31 ± 2.85	22.15 ± 3.89	18.04 ± 7.13
*F* calculation	1.36	5.49	3.91	2.77
*P*	.25	.001*	.01*	.04*
**How many years has s/he been working in this department?**				
0–4	35.05 ± 7.02	7.44 ± 3.33	18.78 ± 5.74	18.88 ± 7.05
5–9	35.59 ± 7.19	7.80 ± 3.84	19.85 ± 3.97	21.26 ± 6.61
10–19	35.19 ± 6.45	6.33 ± 3.99	19.89 ± 5.39	19.78 ± 6.58
20–35	38.75 ± 7.85	4.58 ± 2.77	21.50 ± 4.07	18.25 ± 7.96
*F* calculation	0.91	3.07	1.05	1.23
*P*	.43	.03*	.36	.29
**Has s/he received any training to work in this department?**				
Yes	36.68 ± 6.71	6.72 ± 4.01	19.48 ± 5.39	18.05 ± 7.39
No	34.63 ± 7.25	7.37 ± 3.55	19.77 ± 4.48	21.31 ± 6.34
*t* calculation	1.69	1.07	0.34	0.49
*P*	.09	.31	.73	.007*
**Has s/he asked to work in this department?**				
Yes	36.22 ± 6.12	6.70 ± 3.93	21.13 ± 3.93	18.13 ± 6.74
No	35.22 ± 7.53	7.28 ± 3.68	18.84 ± 5.15	20.73 ± 7.01
*t* calculation	0.77	0.48	0.17	0.74
*P*	.42	.39	.009*	.04*
**Is s/he satisfied with working in this department?**				
Yes	37.33 ± 6.57	6.02 ± 3.15	20.48 ± 4.89	17.17 ± 5.46
No	31.54 ± 5.48	9.72 ± 3.92	17.90 ± 4.32	25.85 ± 6.66
*t* calculation	4.84	5.71	2.86	7.79
*P*	.003*	.05	.04*	.11
**Approximately how many patients are looked after in each duty?**				
0–4	32.89 ± 5.51	8.83 ± 3.63	18.50 ± 3.41	22.50 ± 6.14
5–9	32.82 ± 5.11	7.24 ± 3.05	19.00 ± 3.18	20.29 ± 5.86
10–19	37.27 ± 7.34	6.66 ± 3.58	19.71 ± 5.53	18.82 ± 6.96
20–40	35.42 ± 8.21	6.21 ± 3.61	20.17 ± 4.64	21.04 ± 7.60
*F* calculation	2.84	2.22	0.51	1.54
*P*	.04*	.08	.67	.21
**Duty at the department**				
Clinical nurse	35.61 ± 7.55	6.99 ± 3.76	19.26 ± 5.18	20.23 ± 7.66
Attendant nurse	39.95 ± 6.08	4.95 ± 2.96	22.95 ± 3.47	16.82 ± 5.75
Intensive care nurse	31.96 ± 5.07	8.54 ± 2.96	18.21 ± 3.06	21.29 ± 5.56
Other	34.58 ± 4.88	8.92 ± 4.52	19.17 ± 5.96	19.58 ± 6.54
*F* calculation	5.47	4.96	4.58	1.81
*P*	.001*	.003*	.004*	.14
**Membership at a national association**				
Yes	37.87 ± 6.54	5.39 ± 4.12	22.30 ± 3.21	18.22 ± 7.50
No	35.20 ± 7.04	7.38 ± 3.61	19.20 ± 4.93	20.05 ± 6.85
*t* calculation	1.67	2.35	2.89	1.15
*P*	.09	.02*	.004*	.25
**Does s/he participate in professional courses/congresses?**				
Yes		6.57 ± 3.64	20.58 ± 4.40	19.85 ± 6.63
No		8.32 ± 3.39	19.85 ± 6.63	19.89 ± 7.09
*t* calculation	2.92	2.54	3.11	0.03
*P*	.004*	.01*	.02*	.97
**What are the future plans about profession?**				
Completing undergraduate study	35.57 ± 7.58	7.43 ± 3.85	18.64 ± 6.64	19.29 ± 8.90
Post-graduate study	37.16 ± 6.78	6.70 ± 3.51	20.65 ± 3.98	19.11 ± 6.91
Becoming an academician	37.00 ± 4.59	5.88 ± 2.94	18.25 ± 5.12	15.38 ± 1.76
Improving myself to be better in my profession	35.64 ± 7.63	6.76 ± 3.94	20.44 ± 4.98	19.20 ± 6.15
I do not have any future plans	32.18 ± 5.98	8.54 ± 3.88	18.14 ± 4.75	23.32 ± 7.45
*F* calculation	2.23	1.45	1.66	2.89
*P*	.06	.22	.163	.02*
**Ideas about the future of the profession**				
Positive	37.05 ± 6.31	5.94 ± 3.40	21.02 ± 5.06	17.22 ± 6.42
Negative	34.47 ± 7.44	8.55 ± 3.73	18.67 ± 4.21	23.21 ± 6.57
*t* calculation	2.06	4.02	2.75	5.06
*P*	.04*	.001*	.007*	.005*
**If the ideas about the future of the profession are positive, why?**				
Educational level has increased	36.00 ± 4.41	5.25 ± 2.76	21.01 ± 5.34	14.83 ± 4.41
The transition of males into the profession	44.50 ± 10.60	2.00 ± 0.01	22.50 ± 4.95	14.50 ± 7.77
*t* calculation	2.13	1.61	0.37	0.09
*P*	.05	.02*	.71	.92
Due importance is not given for the profession	33.68 ± 6.56	8.42 ± 3.07	19.26 ± 3.19	23.68 ± 6.98
The workload is too much	33.11 ± 7.86	10.11 ± 3.75	18.78 ± 4.29	24.22 ± 4.49
Uncertain job description	33.50 ± 10.37	8.75 ± 4.78	19.25 ± 4.85	22.50 ± 6.13
Lack of employees	45.50 ± 9.19	7.50 ± 2.12	16.50 ± 0.71	26.50 ± 3.53
Training is not adequate	44.67 ± 2.88	2.67 ± 0.57	22.33 ± 1.52	13.67 ± 1.15
Being considered as assisting employees	37.00 ±	5.00 ±	22.00 ±	15.00 ±
*F* calculation	2.18	2.47	0.83	2.038
*P*	.08	.05	.53	.11

The differences were also significant between having children, choosing and preferring their professions willingly, reasons for choosing this profession, the love for their profession, service years, and working duration at their current departments, the duty in their current departments, membership to a national association, and participation to congresses, their ideas about the future of their profession, and their average scores in the desensitization sub-dimension of the burnout scale (*P* < .05). In the present study, it was determined that the nurses who worked without liking their jobs and the nurses who stated the reason for choosing their jobs as “financial gains” experienced higher rates of desensitization. It was also determined that the nurses who worked between 5 and 9 years in their departments, who were not members of any professional associations, who did not participate in congresses, and who had negative thoughts about the nursing profession had higher scores in the desensitization sub-scale (Table 4).

When the study data were analyzed in terms of gender in the present study, it was determined that the PA and EB Score Averages of the female nurses were higher than those of the male nurses, and the desensitization scores were lower than male nurses (Table 4).

The difference between doing their profession willingly and PA scores was significant, and it was determined that the nurses who did their jobs willingly had higher PA scores. It was observed that the nurses who were members of associations, who participated in congresses, and who had positive thoughts about the future of their professions had higher PA score averages. In addition, the PA Average Scores of the nurses whose service years were between 20 and 35 years were higher when compared to other nurses (Table 4).

The difference between the educational status of the nurses and their EB score averages was significant (*P* < .05). It was determined that the nurses who were associate degree graduates had higher EB scores. It was observed that the EB sub-dimension scores of the nurses who did not choose their professions willingly and whose reason was financial gains for the preference of their profession were higher. It was also observed that the nurses who had service years between 5 and 9 years, who did not like their professions, and who did not receive any training about their departments experienced EB at higher levels. A significant difference was detected in the present study between making future plans about their professions, their ideas about the future of their professions, and EB (*P* < .05); and the EB scores of the nurses who did not have any future plans about their professions, and who had negative thoughts about the future of their professions were found to be higher (Table 4).

The comparison between the self-realization and burnout levels of the nurses is given in Table 4. As a result of the statistical analyses, significant differences were found among the income status, working willingly in their professions, reasons to choose their professions, the love for their professions, satisfaction in their departments, the number of the patients they look after during duty, the duty in their departments, the participation status in professional congresses, the thoughts about the future of their professions, the self-realization scale scores of the nurses (*P* < .05). It was determined that the nurses whose income status was good, those who did their professions willingly, and those who stated that they chose their jobs willingly had higher scores in the scale. In addition, the self-realization scale scores of the nurses who were satisfied from working in their departments, who looked after approximately 10 to 19 patients during their duties, and who worked as Attendant Nurses in their departments were higher than those of the other nurses. Meanwhile, it is noteworthy that the scores of the nurses who had positive thoughts about the future of their professions were higher than the nurses who had negative thoughts about the future of their professions.

As shown in Table 5, a significant difference was detected between the self-realization scores and burnout sub-scale score averages of the nurses, who participated in the study (*P* < .05).

**Table 5:** Examining the relation between the self-realization scale scores and burnout sub-scale scores of the nurses.

Subscales of self-realization scale		Sum of squares	Degree of freedom	Mean square	*F*, *P*
Personal Accomplishment Sub-scale	Between groups	9.347	4	2.337	*F* = 8.850
	Within groups	34.587	131	.264	*P* = *.000
	Total	43.934	135		
Desensitization Sub-scale	Between groups	1.920	4	.480	*F* = 2.666
	Within groups	23.580	131	.180	*P* = *.035
	Total	25.500	135		
Emotional Dimension Sub-scale	Between groups	10.153	4	2.538	*F* = 5.210
	Within groups	63.817	131	.487	*P* = *.001
	Total	73.971	135		

* *P* < .05.

## 4. Discussion

In this section, the relation between the self-realization score averages, the relation between these scores and the independent variables, and the relation between the self-realization score averages and the burnout sub-dimension score averages are discussed in the light of the findings obtained in the present study.

### 4.1. The self-realization score averages of the nurses, and the relation between these scores and independent variables

In the present study, it was determined that the self-realization score average of the nurses was 32.93 ± 6.43 (min.: 13; max.: 52) (Table 3). Onkun (2014) conducted a study and examined the healthy lifestyles of nurses, and reported that the self-realization score average of the nurses was 26.5 ± 4.96.^[17]^ Şimşekoğlu and Mayda (2016) reported that the self-realization score average of the nurses was 35 ± 0.4.^[18]^ In our study, the scale item “*I am proud of my accomplishment*” had the lowest mean value, and the item “*I know my strong and weak sides*” had the highest mean value. The fact that the nurses received the lowest scores in “*I am proud of my accomplishment*” may be associated with the intense workloads, insufficient rewarding, and motivation sources; and the fact that the “*I know my strong sides and weak sides*” item received the highest score may be associated with the positive effects of the training they receive on self-realization.

No significant differences were detected between the age, marital status, self-realization scores, and burnout sub-scale scores of the nurses. However, the self-realization score averages of the nurses who were above 35 years of age were found to be higher. It is considered that there were no significant differences in some variables in the present study because of the limited number of nurses; for this reason, the present study may be repeated with a greater number of nurses. In the study conducted by Onkun (2014) on healthy lifestyles of nurses, it was found that the nurses who were married had higher self-realization score averages than the nurses who were not married.^[19]^ Again, similar to this study, Pirinççi et al (2008) conducted a study and found that married academic staff had higher self-realization score averages than non-academic staff.^[19,20]^ In another study which examines the level of healthy living behavior of Physical Education Teachers, it is seen that Çimen and Kilinç (2017) did not differ significantly in terms of the average scores of marital status variable.^[21]^ In our study, it was observed that there were no significant differences between training and self-realization scores; however, it was found noteworthy that the score averages of the nurses who had Undergraduate Degree was higher (Table 4). Ünsar et al conducted another study,^[19,20]^ and similar to our study, they reported that training did not affect self-realization score averages; however, the self-realization scores of the nurses who had postgraduate degrees were higher. A significant difference was detected between the income status and self-realization score averages of the nurses. The self-realization score averages of the nurses who had good income levels were higher (Table 4). Pirincci et al^[19]^ conducted a study and reported that the self-realization score averages of the academic staff who had high income status were higher.

The self-realization score averages of the nurses, who participated in courses/congresses, who were happy to work in their departments, who loved their professions and who chose their professions willingly, were found to be higher (Table 4). Similar to the results of our study, Ünsar et al (2011) conducted a study and found that the self-realization score averages of the nurses who did their professions willingly were higher.^[19,20]^ In common with this, Arslan (2017) also found out that the teachers who love their jobs more have a higher level of self-actualization compared to the teachers who love their jobs less.^[22]^ In another study, it was reported that 22% of the nurses did not love their professions, and that the professionality levels of those who loved their professions were higher than those who had lower professionality levels.^[23]^

In the present study, it was found that the self-realization score averages of the nurses who worked as Attendant Nurses in their departments were highest, and that of those who worked as Intensive Care Nurses were lowest (Table 4). In the study conducted by Wei Lun Lee et al (2011), whose results were not similar to ours, it was reported that there was a significant difference between the self-realization levels of the nurses, and that the administrative nurses had lower self-realization scores, which was associated with the fact that they had heavy work stress and responsibilities.^[24]^ Onkun et al^[17]^, who found similar results with our study, reported that the self-realization score averages of the nurses, who worked as attendant nurses in their department, was higher.

### 4.2. The burnout sub-dimension score averages of the nurses and its relation with independent variables

When we analyzed the burnout levels of the nurses in 3 sub-dimensions, which were *Emotional Burnout*, *Personal Accomplishment*, and *Desensitization*, it was determined that as the score that was obtained from PA sub-dimension increased, the burnout level decreased; and as the score received from the EB and desensitization sub-dimension increased, the burnout level also increased. The EB score average of the nurses was found to be 19.81 ± 6.99; their desensitization score average was 7.07 ± 3.75; and their PA score average was 19.65 ± 4.87 (Table 3). The difference between gender, desensitization, and EB sub-dimension average scores was insignificant; however, the difference between the PA dimension was found to be significant. It was found that the score averages of the female participants in EB and PA sub-dimension were higher compared to those of the males (Table 4). Kocabiyik and Çakici^[25]^ reported that male nurses experienced less EB compared to females. In the present study of ours, although no significant differences were detected between the marital status and burnout sub-dimension averages, in another study, it was reported that the EB score averages and PA score averages of married nurses were higher.^[26]^

Although there were no significant differences between the ages of the nurses and their burnout levels in the present study, Kaya et al (2010) reported that the EB score averages of the nurses decreased as their age furthered.^[27]^ However, in our study, as the service years of the nurses increased, their PA scores also increased, and EB and desensitization score averages decreased. It was observed that as the total service years increased, the burnout levels decreased. In this respect, it may be claimed that, parallel to the increase in the service years, nurses feel more competent and successful in their profession. The EB score averages of the nurses who worked in the same department for 20 to 35 years were found to be lower. The PA score averages of the nurses who chose their profession with their own will were higher and their EB score averages were lower. And it was determined that the nurses who were satisfied with their departments had higher self-realization scores, and their PA score averages were higher.

Nantsupawat et al confirmed in their research that better work environments are associated with lower job dissatisfaction, burnout, and turnover intention.^[14]^

A significant difference was detected between the burnout scale sub-dimension score averages and their choosing and doing this profession willingly. The EB and desensitization sub-dimension averages of the nurses who loved their professions were lower, and their PA and self-realization scores were higher (Table 4). In another study, it was reported that the nurses who chose their professions willingly had lower EB scores than the other nurses.^[28]^ In the present study, it was determined that the PA average scores of the nurses who considered the reason for preferring this profession as financial gains were lower, and their EB and desensitization score averages were higher. The self-realization levels of the nurses who worked as Attendant Nurses in their departments were higher, and parallel to this, their PA score averages were also higher and the desensitization scores were lower (Table 4). Since Attendant Nurses may be appointed in their departments at the end of a long service year, the fact that nurses who have more than 1 service year have higher self-realization and lower burnout levels coincides with this conclusion. In the present study, significant differences were detected between the course/congress participation, membership at an association, PA averages, and desensitization score averages of the nurses; and it was determined that the PA score averages of these nurses were higher and desensitization scores were lower. The EB score averages of the nurses, who did not have any plans about the future of their professions, and who had negative thoughts about their professions, were found to be higher. It was observed that there was a significant difference between the PA score averages and self-realization score averages of the nurses, who thought positively about the future of their professions (Table 4); and it was determined that the PA and self-realization score averages of the nurses, who had positive thoughts about the future of their profession, were higher.

## 5. Conclusion

Self-realization is perceived as a motivation that manages the behaviors of humans as well as a development level to be reached. In the present study and in some of previous studies, it was determined that the self-realization scores of the nurses, who had postgraduate education and who participated in courses and congresses were high, and the burnout levels were low; for this reason, nurses should be encouraged to receive postgraduate education, to participate in vocational training activities, to attend courses/congresses, etc. Additionally, it may be recommended that the nurse candidates are provided with more detailed guidance about their future professions before they start undergraduate degree education. Organizing in-service training activities and providing support by administrators for the purpose of increasing the motivation and personal development of nurses:

- Organizing courses for personal and professional development in the institution (art, music, handcrafts, English, etc).
- Celebrating the birthdays of working nurses (with text messages and e-mails), giving tickets for cinema, theater, or social activities, etc.

When the fact that experienced nurses feel more adequate and successful in their profession is considered, it would be useful to organize orientation programs through training activities and to create a team that would provide counseling services for nurses, and help them to improve their scientific and professional knowledge to develop their feelings in terms of trust and belonging to their new institutions. It would be useful to create a proper environment for the solution of psychological problems of nurses (psychological guidance); and when burnout that stems from the department they work occurs, it would be useful to transfer them to different departments where they might be more productive.

## Author contributions

**Conceptualization:** Senay Cetinkaya, N. Ecem Oksal Gunes.

**Data curation:** N. Ecem Oksal Gunes.

**Formal analysis:** Senay Cetinkaya, N. Ecem Oksal Gunes.

**Funding acquisition:** Senay Cetinkaya, N. Ecem Oksal Gunes.

**Investigation:** Senay Cetinkaya, N. Ecem Oksal Gunes.

**Methodology:** Senay Cetinkaya, N. Ecem Oksal Gunes.

**Resources:** Senay Cetinkaya, N. Ecem Oksal Gunes.

**Software:** Senay Cetinkaya, N. Ecem Oksal Gunes.

**Supervision:** Senay Cetinkaya.

**Validation:** Senay Cetinkaya, N. Ecem Oksal Gunes.

**Visualization:** Senay Cetinkaya.

**Writing – original draft:** Senay Cetinkaya, N. Ecem Oksal Gunes.

**Writing – review & editing:** Senay Cetinkaya.
